# Global Arts Engagement Inequalities in and Outside School: Analyses of 441,183 15‐Year‐Olds Across 73 Countries

**DOI:** 10.1111/nyas.70151

**Published:** 2025-12-09

**Authors:** Hei Wan Mak, Nisha Sajnani, Nils Fietje, Daisy Fancourt

**Affiliations:** ^1^ Department of Behavioural Science and Health, Institute of Epidemiology & Health Care University College London London UK; ^2^ Program in Drama Therapy, Jameel Arts & Health Lab New York University New York New York USA; ^3^ Behavioural and Cultural Insights Unit WHO Regional Office for Europe Copenhagen Denmark

**Keywords:** arts engagement, global inequalities, meta‐analysis, multilevel modeling, PISA

## Abstract

Participation in and enjoying arts and creative activities is a United Nations human right, offering significant benefits, particularly for young people. However, past research, predominantly from Western countries, has shown that many young people do not engage in the arts and that such engagement is socially patterned, yet research gaps remain. It is unclear whether this pattern is also observed in other parts of the world and whether it is persistent across both in‐school and out‐of‐school contexts in different countries. We analyzed data from the OECD Programme for International Study Assessment (PISA), which surveyed 441,183 15‐year‐olds across 73 countries and found substantial variation in engagement rates. Three key engagement patterns were identified. (1) Countries with higher in‐school engagement rates also had higher out‐of‐school engagement rates. (2) Most students engaged more in the arts in school than out of school. (3) Individual‐, school‐, and country‐related factors may influence engagement, with a strong social gradient, especially for out‐of‐school engagement. Schools hold the potential to equalize engagement in and outside school and thus reduce cultural, health, and academic inequalities. This aligns with Sustainable Development Goal 3: promoting wellbeing for all, and is relevant across multiple sectors and countries worldwide.

## Graphical abstract

This paper explored patterns and factors of arts and creative engagement in and outside school among young people across 73 countries. Using data from the OECD PISA, we found that countries with higher in‐school engagement rates also had higher out‐of‐school engagement rates and that factors of engagement operate across different levels, with a stronger social gradient for out‐of‐school engagement.

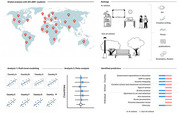

## 1 Introduction

Arts and creative engagement (such as playing a musical instrument, arts and crafts, drama) is increasingly recognized as a key health behavior, with wide‐ranging benefits for mental and physical health [1, 2, 3]. A substantial body of work demonstrates the benefits of arts and creative engagement for young people, including improvements in school attendance, classroom behaviors, psychological and physical wellbeing, social interactions, emotional competence, and prosocial behavior [1, 3]. Yet, despite it being a United Nations human right for everyone to “participate in the cultural life of the community” and “to enjoy the arts” [4], recent epidemiological research suggests that many young people are not engaging in the arts at all. For example, in the UK, fewer than half of young people aged 16−24 engage in cultural activities (e.g., visiting museums and galleries), and one in four reports never participating in any artistic activities [5]. This figure is even higher in Canada, where 36% of young people aged 15−24 report not engaging in any arts activities, although attendance at cultural events is higher (82% attending at least once in a year) [6]. However, comprehensive data on youth engagement rates are predominantly available only in a limited number of Western countries, leaving it unclear whether these disparities are similarly observed in other parts of the world.

In addition, there are stark inequalities in which young people do engage in the arts. In the UK and Ireland, studies show that young people from ethnic minority backgrounds, males, those in lower socioeconomic positions, and individuals living in more deprived areas are less likely to engage with the arts [7, 8]. However, when this is broken down by where people engage, the results are more nuanced. These same analyses demonstrated that out‐of‐school engagement (when young people take part in activities outside of the formal or compulsory classes, e.g., extra‐curricular activities) is more starkly patterned by socioeconomic position than in‐school engagement (where schools provide classes or programs as part of the national curriculum, offering accessible arts materials such as paints, musical instruments and books, and organizing subsidized arts school trips) [7]. However, the extent to which the social gradient of engagement persists across both in‐school and out‐of‐school contexts in different countries remains insufficiently explored, and there has also been limited consideration of how participation in versus outside school may also be affected by other factors. For example, qualitative research in Singapore highlighted the influence of both intrinsic factors (e.g., personal passion for the arts) and extrinsic factors (e.g., young people's social milieu) [9], and it remains likely that country‐level factors such as government funding for education and country GDP influence opportunities for schools to provide arts [10].

The most recent OECD Programme for International Study Assessment (PISA) 2022 offers novel opportunities to expand our understanding of the patterns and predictors of young people's arts and creative engagement both in and out of school across countries. Notably, it allows researchers to examine how engagement is influenced by individual‐, school‐, and country‐related factors, potentially highlighting structural inequalities that could be contributing to and reinforcing disparities in health and educational outcomes. Therefore, in our paper, we analyzed data from over 441,000 young people aged 15 in 73 countries. We hypothesized that a social gradient exists in arts and creative engagement, with out‐of‐school engagement showing a stronger inequality. Our objectives were to (1) compare engagement rates in and outside of school across countries, and (2) identify key factors influencing engagement in both contexts.

## 2 Methods

### 2.1 The PISA Study

The PISA study assesses 15‐year‐olds in school in grade 7 or higher [11]. In most participating countries/regions, these students are approaching the end of compulsory schooling. PISA surveys take place every 3 years, with the first survey taking place in 2000. They collect cross‐sectional data from students, teachers, schools, and parents on students’ sociodemographic backgrounds, wellbeing, school and learning environments, and other contextual factors that may influence their academic experience and outcomes. Particularly, the surveys focus on and assess students in three core subjects: reading, mathematics, and science, and how students can apply the knowledge and skills they learned and practiced at school in specific or real‐life situations and challenges. PISA also conducts assessments of cross‐curricular competencies, including general problem‐solving competencies and financial literacy [11].

In our study, we analyzed data from the latest wave of PISA, the PISA 2022 survey, which includes a new “creative thinking” module. The creative thinking module explores students’ participation in creative activities in school and outside school, their performance and attitudes associated with creative thinking, and opportunities to engage across schools and countries [12]. Around 690,000 students from 81 participating countries or regions completed the assessment in the PISA 2022, representing 29 million 15‐year‐olds in the schools [11, 12]. For our analysis, 441,183 students from 73 countries or regions completed data on their engagement with arts and creative activities inside and outside school. Of them, 234,872 students from 50 countries completed data across all measures. These countries include: Albania, Argentina, Austria, Belgium, Brazil, Bulgaria, Canada, Chile, Colombia, Croatia, Czech Republic, Denmark, Dominican Republic, El Salvador, Estonia, Finland, France, Georgia, Germany, Greece, Hungary, Indonesia, Italy, Jamaica, Kazakhstan, Latvia, Lithuania, Malaysia, Malta, Mexico, Mongolia, Netherlands, Panama, Peru, Philippines, Poland, Portugal, Republic of Moldova, Romania, Serbia, Slovak Republic, Slovenia, Spain, Switzerland, Thailand, Türkiye, Ukrainian regions (18 of 27), the United Kingdom (UK), Uruguay, and Uzbekistan.

### 2.2 Measures

#### 2.2.1 Arts and Creative Engagement

Five arts and creative activities were asked about: (1) art classes/activities (e.g., painting, drawing); (2) creative writing classes/activities; (3) music classes/activities (e.g., chorus, band); (4) dramatics, theater class/activities; and (5) publications (e.g., newspapers, yearbooks, literary magazine). Students were asked how often they participated in each of these activities in school and outside of school. Response items included never or almost never, about once or twice a year, about once or twice a month, about once or twice a week, every day or almost every day, and not available at school/outside school. In our study, we generated two specifications to cross‐validate our results. First, in our main analysis, a binary variable was generated (1 = engaged vs. 0 = never or almost never or not available at school/outside school). Second, we repeated the analysis by removing responses for the item “not available at school/outside school” as a robustness check.

#### 2.2.2 Factors of Engagement

To identify key enablers and barriers to engagement in school and outside school, we considered multilevel factors. For *individual‐related predictors*, we considered students’ gender (female vs. male), country of birth (native born vs. immigrant), parents’ education level (up to lower secondary education, upper secondary education, degree or above), home possessions (Table S1 for a list of items; a total of 19 response items was summed, averaged, and then standardized within each country), arts resources (Table S1; five response items were summed, averaged, and then standardized within each country), and openness to art and reflection (Table S1; five response items were summed, averaged, and then standardized within each country).

For *school‐related predictors*, we considered creative school and classroom environment (Table S1; six response items were summed, averaged, and then standardized within each country), the location of the school (in rural area, in town, in city), and the type of school (private school that is managed by nongovernment organization, e.g., a church, trade union, business or other private institution, vs. public school that is managed by a public education authority, government agency, or governing board appointed by the government or elected by public franchise). The location and the type of school were answered by the school principal or designate.

For *country‐related predictors*, we considered the world happiness index (standardized) [13], Gini income inequality index (standardized) [14], log GDP in capita [15], and government expenditure on education (total, % of government expenditure; standardized) [16]. These country‐related predictors were derived from the World Happiness Report and the World Bank.

### 2.3 Analysis

Our analyses focused on two aims: (1) to explore the prevalence and patterns of arts and creative engagement among young people inside and outside school, and (2) to identify predictors of engagement. To identify key predictors, we used multilevel logistic models. We fitted a two‐level model to account for the nested structure of the PISA data, as students living in the same country were likely to have similar experiences, attitudes, and behaviors to each other than to those living in another country. In the model, we allowed for the estimation of a random slope for gender as we assumed that the association between gender and engagement varied across countries. Odds ratios and 95% confidence intervals (CIs) are reported to present the direction and magnitude of the association between predictors and engagement. Senate weights provided by PISA were applied in the analysis to ensure that each country contributes equally to the results. This means that, for instance, the estimated impacts for Panama are weighted the same as those for Canada, allowing for a balanced comparison between countries [17].

Four sensitivity analyses were conducted. First, given that some education is free to parents, while other education requires fee payment, we restricted the sample to those who were in public school only (*N* = 194,753). Second, we categorized those 50 countries by their economic development (according to the World Bank) [18] and grouped them into either high‐income countries or middle‐income countries. Logistic regression was then conducted separately for each country‐income category. This analysis controlled for country of residence to capture sociocultural variances across countries and omitted country‐related predictors. Third, we repeated the main analysis by omitting the engagement response option “Not available” to restrict the sample to those where activities were available and students were aware of them (*N* = 199,739).

Following these analyses, we performed a meta‐analysis on individual‐ and school‐related predictors that showed heterogeneity in the above analyses. Using all available data, we first analyzed the associations between the predictors and engagement rates for each country using logistic regression. Study‐specific estimates (odds ratios and standard errors) were then pooled into a meta‐analysis using the random effects model to estimate the overall effect sizes for outcomes (*N* of study = 66 countries; sample *N* = 308,008). Heterogeneity was examined by computing I2, H2, and T2 statistics. I2 is the percentage of variability in the effect size that is caused by between‐study heterogeneity, rather than by sampling error. The H2 statistic describes the ratio of the observed variation and the expected variance due to sampling error. The T2 statistic is the variance in proportions across countries and is an indicator of cross‐national heterogeneity [19, 20, 21].

Missing data were handled using listwise deletion (30.2% of participants did not complete individual‐ and/or school‐related data). The risk of multicollinearity was minimal (mean VIF = 1.77).

## 3 Results

### 3.1 Descriptive Patterns of Engagement

**FIGURE 1:**
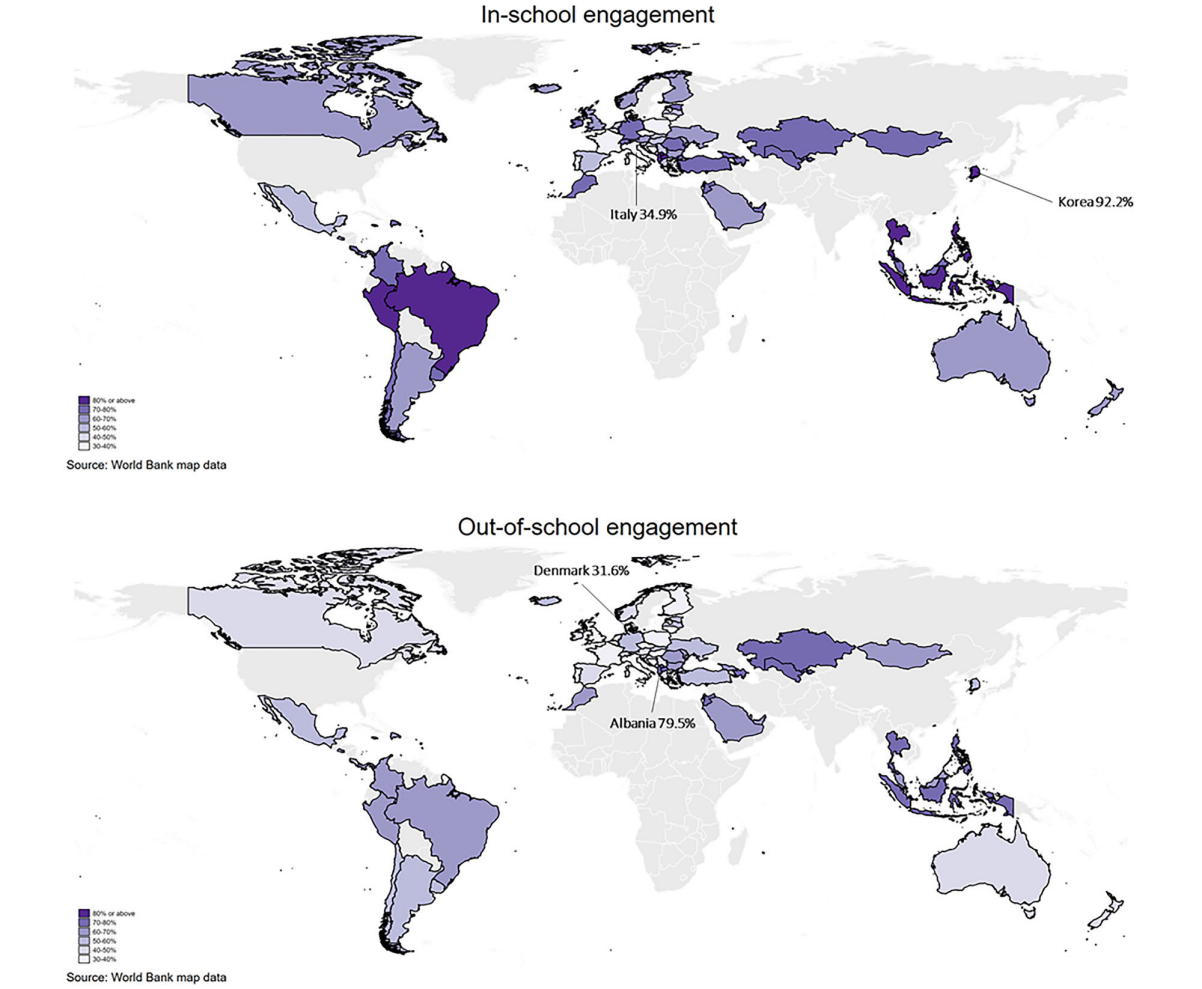
| Patterns and rates of in‐school and out‐of‐school arts and creative engagement (*N* = 441,183 from 73 countries or regions). For some countries/regions, there are slight discrepancies between the World Bank map data and data collected in PISA: Chinese Taipei is not shown on the map as it is not listed as an independent entity in the World Bank map data. Baku (Azerbaijan), Brunei Darussalam, Palestinian Authority, and Ukrainian regions (18 of 27) appear as Azerbaijan, Brunei, Palestine, and Ukraine in the map.

Students in Korea, Thailand, the Philippines, Indonesia, and Albania had the highest in‐school engagement rate out of all 73 countries/regions (Figure 1 and Table S2). In particular, 92.2% of Korean students reported engaging in arts and creative activities in school, followed by 86.4% in Thailand, 85.7% in the Philippines, 84.6% in Indonesia, and 84.3% in Albania. In contrast, engagement rates were lower in some countries, with only 34.9% of students in Italy reporting engagement, followed by 35.6% in Portugal, 35.8% in the Czech Republic, and 35.9% in Poland.

Countries that had a higher in‐school engagement rate generally showed a higher out‐of‐school engagement rate (Figure 1 and Table S2). For example, Thailand (78.6% engaged outside of school), the Philippines (78.1%), Indonesia (76.4%), and Albania (79.5%) had high engagement outside school as well. Similarly, countries with a lower in‐school engagement rate tended to have a lower out‐of‐school engagement rate, such as Poland (31.9%), the Czech Republic (36.3%), Portugal (33.8%), and Italy (31.7%). However, there were notable exceptions. In Korea, Chinese Taipei, and Macao (China), over 82% students engaged in arts and creative activities in school, yet only 41.4−52.6% did so outside school. Across all countries, engagement was higher in school than outside school, except in the Czech Republic (although the difference was minimal).

**FIGURE 2:**
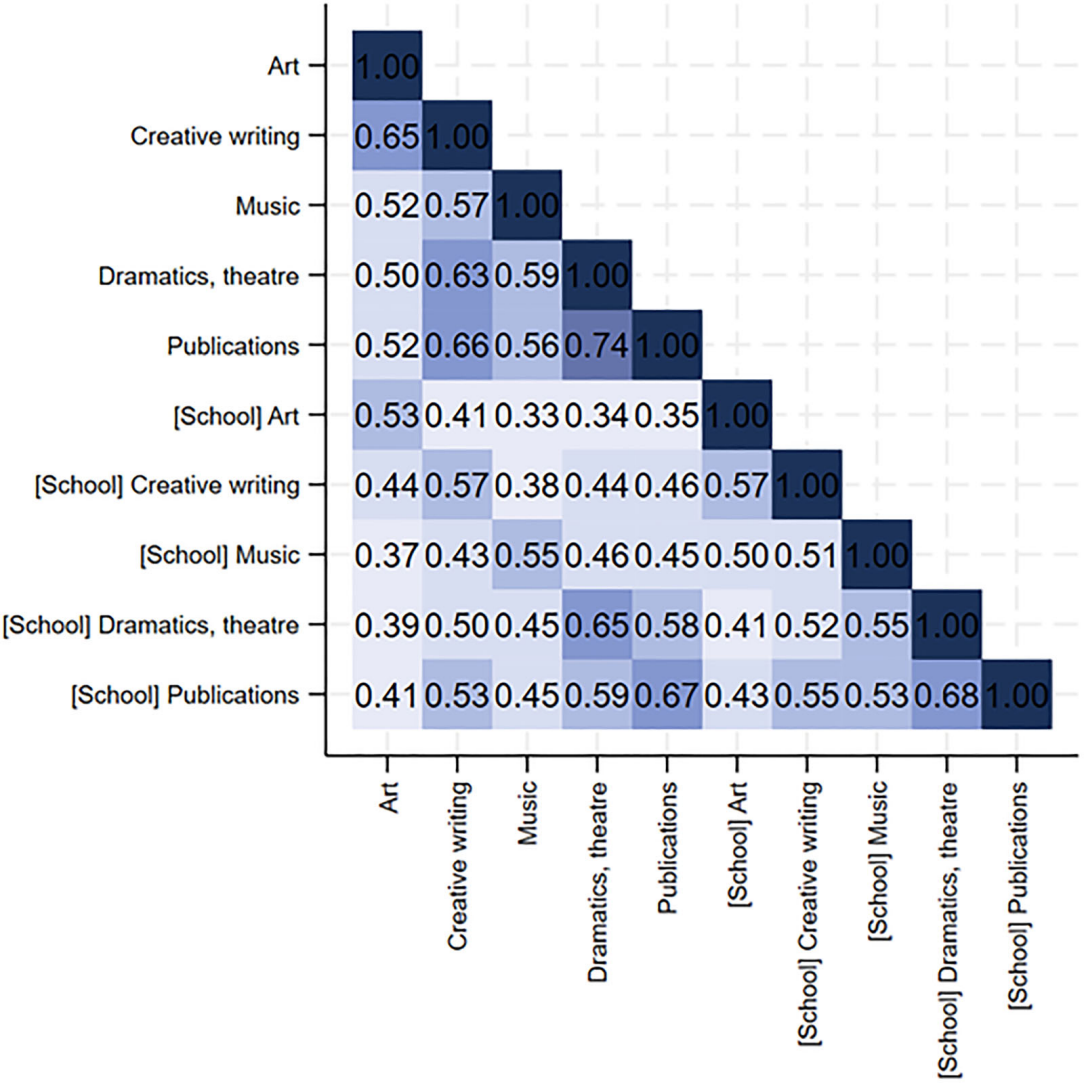
| Matrix of correlation between in‐school and out‐of‐school arts and creative engagement.

The correlations between different types of arts and creative activities, both in‐school and out‐of‐school, were moderate to high. The strongest correlation was observed between dramatics/theater and publications (*r* = 0.68 for in‐school engagement and *r* = 0.74 for out‐of‐school engagement) (Figure 2). Students who frequently engaged in dramatics/theater in school were also more likely to report engaging frequently in the same activity outside school (*r* = 0.65). A similar pattern was observed for publications (*r* = 0.67) and creative writing (*r* = 0.57).

### 3.2 Predictors of Engagement

Out of the total 73 countries, 50 had data on individual‐, school‐, and country‐related predictors. In this reduced analytical sample, 53.0% of participants were female, 93.2% were native born, and 49.0% had parents with a degree or above (Table 1). On average, 39.6% of students attended school in a city, and 83.9% were in public schools.

**TABLE 1:** Descriptive statistics of analytical sample (*N* = 234,872 from 50 countries or regions).

	% or Mean (SD)
Male	47.0%
Female	53.0%
Immigrant	6.78%
Native born	93.2%
[Parent] Up to lower secondary education	11.0%
[Parent] Upper secondary education	39.9%
[Parent] Degree or above	49.0%
Home possessions, ranging from 0 to 2.87	1.02 (0.34)
Arts resources, ranging from 0 to 3	1.12 (0.81)
Openness to art and reflection, ranging from 1 to 4	2.66 (0.73)
School in rural area	30.3%
School in town	30.1%
School in a city	39.6%
Private school	16.2%
Public school	83.9%
Creative school and classroom environment, ranging from 1 to 4	2.75 (0.64)
World happiness index, ranging from 4.74 to 7.82	6.20 (0.67)
Gini income inequality index, ranging from 24.1 to 54.8	34.2 (6.89)
Log GDP in capita, ranging from 7.73 to 11.4	9.67 (0.87)
Government expenditure on education (total, % of government expenditure), ranging from 7.11 to 24.1	12.3 (3.98)

#### 3.2.1 Individual‐Related Predictors

**FIGURE 3:**
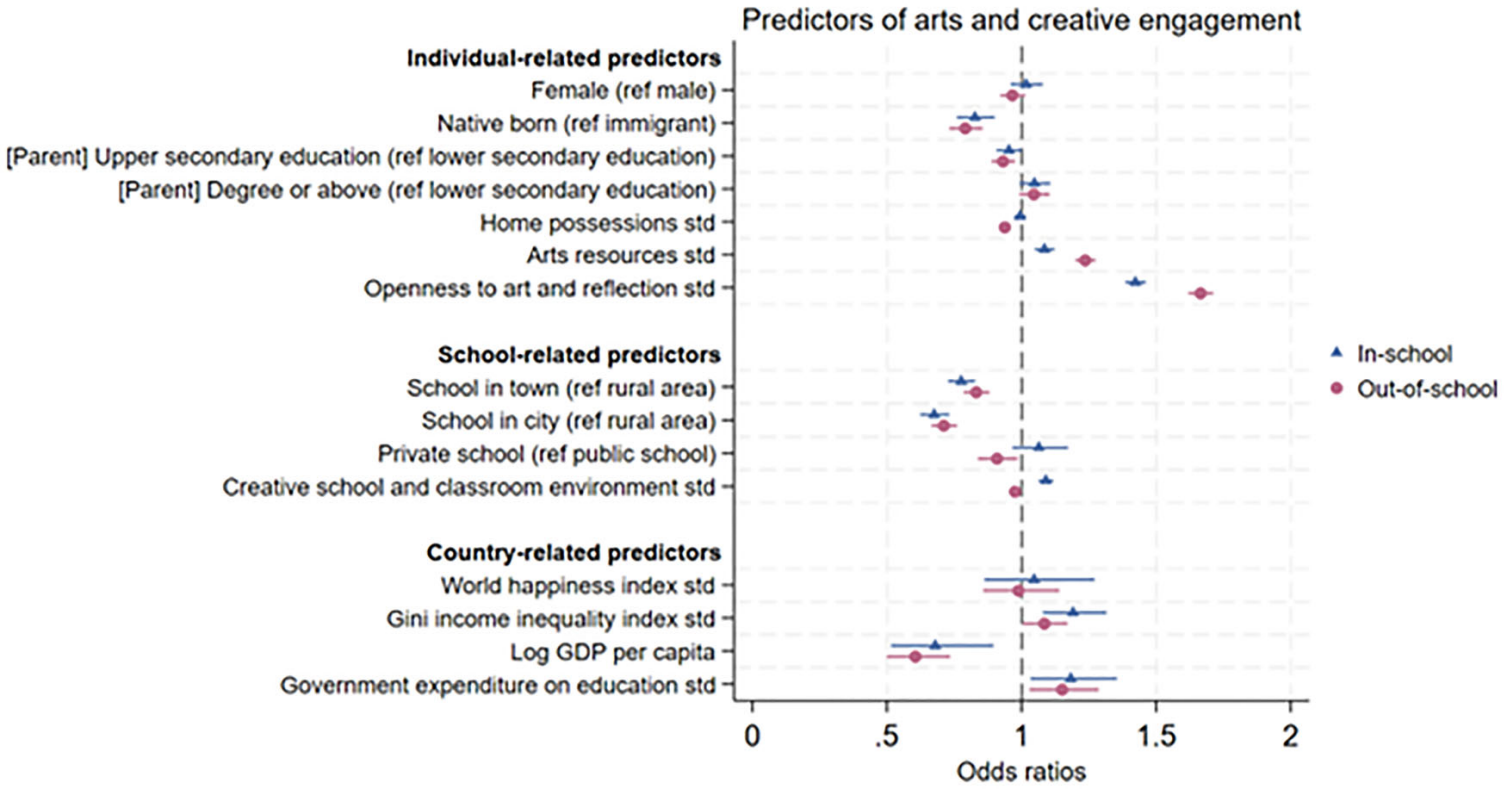
| Predictors of in‐school and out‐of‐school arts and creative engagement, estimates from multilevel logistic regression models (*N* = 234,872; 50 countries or regions). Odds ratios and 95% CI are presented.

There was evidence of a social gradient for both in‐school and out‐of‐school engagement, but more so for the latter (Figure 3 and Table S3). For in‐school engagement, students with more arts resources at home (OR = 1.08, 95% CI = 1.05, 1.12), and those who had greater levels of openness to art and reflection (OR = 1.42, 95% CI = 1.39, 1.46) had higher odds of engaging in arts and creative activities in school. However, those who were native born (OR = 0.83, 95% CI = 0.76, 0.90) were less likely to engage in arts and creative activities in school. For out‐of‐school engagement, a similar pattern was observed, although certain factors were uniquely associated with this type of engagement (Figure 3 and Table S3). Students whose parents had an upper secondary education level had 6.92% lower odds of engaging in arts and creative activities outside school, compared to those whose parents had an education level up to lower secondary. Further, there was a negative association between home possessions (OR = 0.94, 95% CI = 0.92, 0.96) and out‐of‐school engagement. Meta‐analysis of the association by country supports this finding, showing a pooled OR of 0.96 and 95% CI between 0.94 and 0.98, whereas no association was found for in‐school engagement (Figure S1A,B).

While no associations were found for gender across countries/regions as a whole, we found some heterogeneity between individual countries/regions (Figure S2). For instance, for both in‐school and out‐of‐school engagement, girls were more likely to engage in arts and creative activities in countries/regions such as Albania, Germany, Romania, the UK, and the Netherlands, whereas countries/regions like Argentina, Chile, Colombia, the Philippines, and Uruguay had more boys engaging in the activities than girls. No significant gender differences were found in Spain and Jamaica for both in‐school and out‐of‐school engagement.

**FIGURE 4:**
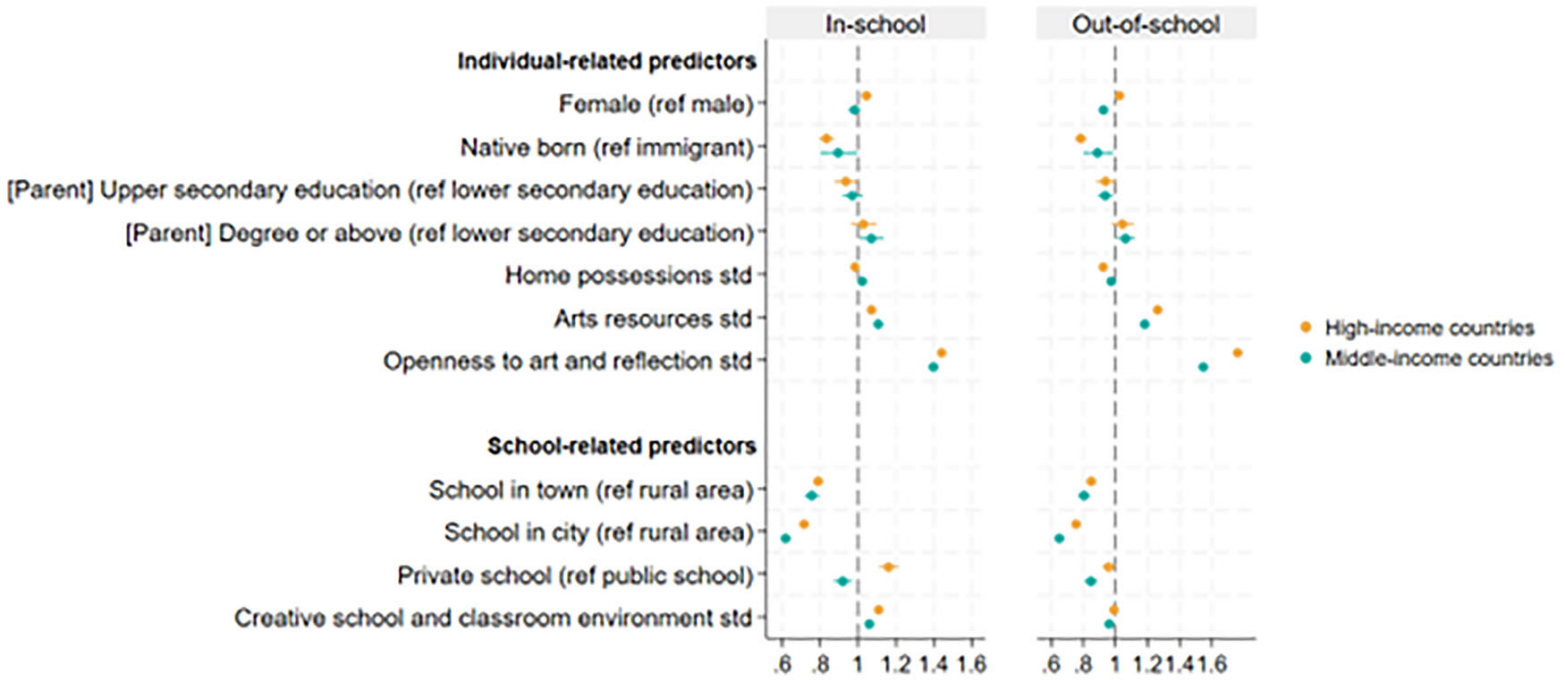
| Predictors of in‐school and out‐of‐school arts and creative engagement by country‐income category, estimates from logistic regression models. Odds ratios and 95% CI are presented. Country‐related predictors were omitted from the models. Models adjusted for country. *N* = 134,012 for high‐income countries/regions, and *N* = 100,860 for middle‐income countries/regions.

When categorizing countries/regions as high‐income or middle‐income, patterns were similar (Figure 4 and Table S3). However, some notable differences were observed: in high‐income countries/regions, girls engaged more in arts and creative activities in school, but there was no difference in out‐of‐school engagement, whereas in middle‐income countries/regions, there was no difference in in‐school engagement, but they engaged less outside of school. Also in middle‐income countries, students whose parents had a degree or above educational level engaged more in the activities both in and outside school. However, the association between parents’ degree and in‐school and out‐of‐school engagement was less substantial in the meta‐analysis (Figure S3A,B).

When excluding the “not available” response option from the engagement variables, the results were materially unchanged (Figure S4 and Table S3). Yet, students whose parents had a degree or above education level engaged more in in‐school activities.

Restricting the sample to students who attended public schools, results were very similar (Figure S5 and Table S3). A notable difference was that those whose parents with a degree or above engaged more both in and outside school. However, boys engaged less outside of school.

#### 3.2.2 School‐Related Predictors

Students who enrolled in schools with a greater level of creative school and classroom environment (OR = 1.09, 95% CI = 1.06, 1.12) had higher odds of engaging in arts and creative activities in school. But an opposite pattern was observed for out‐of‐school engagement (OR = 0.98, 95% CI = 0.95, 1.00) (Figure 3 and Table S3), and was more prevalent in middle‐income countries/regions (Figure 4 and Table S3) and public schools (Figure S5 and Table S3).

Compared to students in rural schools, those in town or city schools were less likely to engage in arts and creative activities both in school (town: OR = 0.78, 95% CI = 0.73, 0.83; city: OR = 0.67, 95% CI = 0.62, 0.73) and outside of school (town: OR = 0.83, 95% CI = 0.78, 0.88; city: OR = 0.71, 95% CI = 0.66, 0.76) (Figure 3 and Table S3). The pattern remained consistent across high‐income and middle‐income countries/regions, as well as in other sensitivity analyses.

In contrast, school types showed variation. Students in private schools engaged less than those in public schools in arts and creative activities outside of school (OR = 0.91, 95% CI = 0.84, 0.99) (Figure 3 and Table S3). This trend was further supported by the meta‐analysis (pooled OR: 0.89, 95% CI = 0.82, 0.96) (Figure S6A) and was more pronounced in middle‐income countries/regions (Figure 4 and Table S3). However, for in‐school engagement, private school students engaged more in high‐income countries/regions but less in middle‐income countries/regions (Figure 4 and Table S3). Additionally, they were more likely to engage when these activities were available in school and they were aware of them (Figure S4 and Table S3). The correlation between school types and in‐school engagement was, however, less supported by the meta‐analysis (Figure S6B).

#### 3.2.3 Country‐Related Predictors

On a country‐level, Gini income inequality index (OR = 1.19, 95% CI = 1.08, 1.32) and government expenditure on education (OR = 1.18, 95% CI = 1.03, 1.35) were positively associated with in‐school engagement, whereas GDP in capita (OR = 0.68, 95% CI = 0.51, 0.90) was negatively associated with such engagement. For out‐of‐school engagement, a similar pattern was observed (Figure 3 and Table S3). Sensitivity analyses showed very similar patterns (Figures S4 and S5; Table S3).

## 4 Discussion

This study explored the prevalence and predictors of in‐school and out‐of‐school arts and creative engagement across 73 countries. Engagement rates vary substantially between countries. For in‐school engagement, Korea has the highest proportion of students engaging in arts and creative activities (92.2%), while Italy has the lowest (34.9%). For out‐of‐school engagement, Albanian students have the highest engagement rate (79.5%), whereas Danish students have the lowest (31.6%). Additionally, we identified three key engagement patterns. First, countries with higher in‐school engagement rates also tend to have higher out‐of‐school engagement rates (e.g., in Thailand, the Philippines, Indonesia, and Albania), while countries with lower in‐school engagement rates tend to have lower out‐of‐school engagement rates (e.g., Italy, Portugal, the Czech Republic, and Poland). This pattern is particularly evident for certain activities such as dramatics/theater, publications, and creative writing. Second, in most countries, students engage more in arts and creative activities in school than out of school, except for the Czech Republic, where the difference is very small. Third, aligning with the results we found for community arts group membership among adults in our previous global study [22], in exploring the predictors of both types of engagement, we found that factors related to individual, school, and country may influence whether students engage in arts and creative activities. A clear social gradient is observed for both types of engagement, particularly for out‐of‐school engagement.

Many of the strongest predictors of arts engagement apply to both in‐school and out‐of‐school engagement. Specifically, students who are native‐born, attend schools in towns or cities, or live in countries with high GDP per capita tend to engage less in both in‐school and out‐of‐school activities. On the other hand, higher engagement rates are found among students with greater access to arts resources, higher openness to arts and reflection, and those living in countries with a higher Gini income inequality index and greater government expenditure in education. Some of these findings align with previous research, especially the greater access to arts resources at home and higher openness to arts and reflection. Drawing upon Bourdieu's cultural capital [23], young people's cultural tastes, preferences, and behaviors are shaped by their families, with those whose upbringing involves more exposure to the arts are more likely to perceive opportunities to engage in these activities. Arts resources and openness to art and reflection can be considered as a form of cultural capital, which include (1) material capital (e.g., classical literature, works of art) and (2) embodied capital (e.g., values, preferences, attitudes toward the arts) [23]. Both of these types of factors are important in both in‐school and out‐of‐school settings across high‐ and middle‐income countries/regions, suggesting the potential universality of cultural capital in influencing young people's engagement.

Another predictive factor is school location, in which we found that engagement rates are higher in students who attend schools in rural areas. This is also reflected in a UK study on an adult sample [24]. While rural areas may have fewer arts and cultural infrastructures and venues or have poorer transportation links to those venues, schools in these areas may offer local‐ or community‐based activities to compensate for this and encourage participation. Further, on a country‐level, more government expenditure in education can ensure (1) well‐funded education systems, (2) high‐quality arts and cultural education curriculum and extra‐curriculum, and (3) more equalized access to education and arts‐related curriculum. For instance, countries including Kazakhstan, the Dominican Republic, Uzbekistan, Malaysia, and Morocco, where around 70% or above of our student samples engage in arts and creative activities inside and outside school, over one‐fifth of the total government expenditure is spent on education in those countries [16].

However, other of the identified shared factors are less aligned with previous studies and may be more surprising. For instance, in opposite to what we found, in the UK and Europe, those who were EU‐born or those who were the ethnic majority in their country tend to have higher engagement rates [7, 25, 26]. Yet, most studies focus on ethnicity/country of birth in adult samples, and some of them show variations in cultural and ethnic heritage and the types of arts and creative activity engagement [27, 28]. Further studies are needed to disentangle the associations between country of birth, ethnicity, and engagement among young people. In addition, countries with higher levels of income inequality and lower GDP per capita, which are associated with greater engagement in the arts among young people, may have their schools focus on arts‐related programs and activities, potentially creating a spillover effect on out‐of‐school engagement.

Our analysis also identified factors that uniquely affect out‐of‐school engagement. For instance, students with a higher level of home possessions, those attending private school, and those in greater creative school and classroom environments tend to engage less in arts and creative activities outside of school. (The finding that children of parents with an upper secondary education engage less was not supported by the meta‐analysis.) However, these patterns do not extend to in‐school engagement. Instead, students exposed to higher levels of creative school and classroom environment are more likely to engage in arts and creative activities in school. Several explanations may account for this. First, more home possessions may create distractions for young people to engage in the arts, and while economic capital allows access to material arts goods, it may not always cultivate motivation or the skills to engage in these activities. Second, students in private schools or those exposed to a creative school and classroom environment may already have sufficient opportunities to engage in arts and creative activities during school hours or through extra‐curricular provision, and parents may feel these activities have already been financially supported through school tuition fees, and hence these children may not participate in additional out‐of‐school activities.

However, it is also important to recognize country‐specific variations. For instance, the negative association between private school and in‐school engagement is more prevalent in middle‐income countries/regions, and an opposite pattern is observed in high‐income countries. Similarly, although gender is not associated with engagement levels in both in‐school and out‐of‐school settings in the main analysis, when separating countries by the country income category, we did find some gender differences: girls engaged in more in‐school activities in high‐income countries/regions [7, 25], but engaged less in out‐of‐school activities in middle‐income countries/regions. But even within each country income category, there are still gender variations in engagement. In addition, although our main findings are largely supported by follow‐up meta‐analyses, they do reveal variations between countries. This country‐specific variation suggests a complex picture of within‐ and between‐country differences in young people's in‐school and out‐of‐school participation in arts and creative activities, and more in‐depth analyses are required to further disentangle persistent and country/cultural‐specific enablers and barriers of engagement.

Our findings lead to three key inferences. First, engagement in the arts and creative activities is not only shaped by individual‐related factors, but also by broader influences at different levels of influence [29]. Notably, out‐of‐school engagement is still influenced by school‐related factors such as the location, type, and environment of schools, and is moderately to highly correlated with in‐school engagement. This highlights the crucial role of schools in providing arts and creative opportunities. Indeed, schools can be a modifiable factor, where government and private sectors can offer funding to ensure more diverse in‐school arts and creative programs delivery and a more balanced curriculum between arts and nonarts subjects and activities. This is supported by our findings, which show that countries that spend a greater proportion of their total government in education have more students engaging in arts and creative activities both inside and outside schools. Second, these factors reflect the importance of students’ capabilities, opportunities, and motivations in encouraging engagement, aligning with the COM‐B model of behavior change [30]. Schools that place emphasis on providing a creative environment, subsidizing cultural trips or musical instruments, and supplying arts tools and resources can enhance students’ artistic skills, while offering social and physical opportunities to participate. Since arts and cultural engagement in adulthood is strongly related to early‐life participation, removing barriers and enhancing enablers for young people can have long‐term impacts throughout their lives. Schools may be the key to this. Third, since research suggests that arts and creative engagement is associated with better academic performance and myriad health benefits in adolescence and across the life‐course [1], equalizing access to and participation in the arts could have the potential to reduce the existing academic and health inequalities.

The present research has many strengths, including a large sample from 73 countries across geographically and culturally diverse regions. Further, the PISA dataset contains standardized questions across countries, which helped minimize measure biases and enable large‐scale cross‐country comparisons in patterns and predictors of arts and creative engagement. The dataset also included rich data on in‐school and out‐of‐school arts and creative engagement and allowed us to explore individual‐, school‐, and country‐related factors of different engagement settings. However, the study is not without limitations. First, due to the cross‐sectional nature of the data, we were not able to establish causal relationships. However, most of the exposures were likely to temporally precede engagement. Second, students with moderate‐to‐severe permanent disability or cognitive, behavioral, or emotional disability might have been currently excluded from the PISA data to ensure validity and comparability [31], and these individuals may experience greater barriers to engagement [32, 33]. Further research is needed to explore whether factors identified in the present survey are also observed in different student demographics. Further, high‐level cultural infrastructure and policy, government expenditures on the arts, and school funding for arts and creative programs are likely to differ across countries, which may affect students’ participation both in and outside school. In‐depth policy analysis is required to evaluate the effectiveness of current arts and cultural policy and implementation of arts education, and how they may influence both in‐school and out‐of‐school engagement.

In addition, there are important challenges when undertaking global research on arts and creative engagement. For instance, culturally specific art forms or local arts practices might not have been captured in the PISA data measures. In some countries, where arts and creative activities are closely integrated into daily life, participation may be under‐represented, leading to lower reported engagement rates. There are also challenges relating to limited in‐depth metrics of engagement, including the duration, intensity, and forms (formally vs. informally or in groups vs. individually) [2]. Yet, focusing on engagement rate (engaged vs. not engaged) can help reduce measurement biases due to differences in definitions or interpretations, which is particularly important in cross‐country comparisons where concepts will naturally be understood differently across cultures. To take this work further, qualitative aspects of engagement, such as students’ attitudes, values, and enjoyment of the arts, could be considered in future work. As with any observational data, self‐reporting bias may exist. As such, it is important to acknowledge that our analysis only presents part of the overall picture of global patterns of engagement among young people inside and outside school.

## 5 Conclusions

Overall, our findings indicate that there is inequitable access to and participation in in‐school and out‐of‐school arts and creative activities globally. Factors influencing arts engagement operate at various levels, and some can be applied to both in‐school and out‐of‐school settings, such as country of birth, arts resources, openness to arts and reflection (individual‐related factors), school location (school‐related factors), GDP per capita, Gini income inequalities, and government expenditure in education (country‐related factors). However, there are also some unique factors influencing particularly out‐of‐school engagement, as well as engagement between countries. Our findings have two important implications. First, schools could be an effective and modifiable target of intervention in encouraging students’ arts and creative engagement, by cultivating their artistic skills, providing social and physical opportunities, and offering resources that foster incentives to engage with behavioral effects that could last beyond school and into adulthood, promoting life‐long engagement in the arts. This aligns with aims to achieve human rights such as the UN's Universal Declaration of Human Rights which states that “everyone has the right freely to participate in the cultural life of the community, to enjoy the arts and to share in scientific advancement and its benefits” [4]. Second, schools hold the potential for a spillover effect, helping to reduce cultural inequalities, especially in out‐of‐school engagement, as well as academic and health disparities, given the link between arts and creative participation and mental and physical health outcomes across the life‐course. This places access to arts education within broader aims to achieve Sustainable Development Goals (SDGs), in particular SDG3: ensuring healthy lives and promoting wellbeing for all at all ages. As such, this topic has importance to multiple sectors and all countries around the world.

## Author Contributions

All authors designed the study. H.W.M. analyzed data and drafted the manuscript. D.F. supported with analytical issues and contributed to the writing. All authors made critical revisions and approved the final manuscript.

## Funding

The study was supported by Jameel Arts & Health Lab Global Population Health Fellowship. This paper was also carried out as part of the EpiArts Lab, a National Endowment for the Arts Research Lab at the University of Florida, which is supported in part by an award from the National Endowment for the Arts (1862896–38‐C‐20). The EpiArts Lab is also supported by the University of Florida, Americans for the Arts, the Pabst Steinmetz Foundation, and Bloomberg Philanthropies.

## Conflicts of Interest

The authors declare no competing interests.

## Ethical Approval Statement

Ethical approval was not required for this study, as it analyzed secondary data that were anonymized and publicly available.

## Supporting information



Supplementary Materials: nyas70151‐sup‐0001‐SuppMat.docx

## Data Availability

Data are publicly available on the OECD PISA database website: https://www.oecd.org/en/data/datasets/pisa‐2022‐database.html#data. All codes used for these analyses are publicly available online: https://osf.io/tw93s/.
