# Supplementary material for: Global Arts Engagement Inequalities in and Outside School: Analyses of 441,183 15‐Year‐Olds Across 73 Countries
Source: Ann N Y Acad Sci. 2025 Dec 9;1556(1):e70151. doi: 10.1111/nyas.70151 (PMC12915476; doi:10.1111/nyas.70151)
Supplement: Supplementary file 1 — Supplementary Materials: nyas70151‐sup‐0001‐SuppMat.docx [file NYAS-1556-0-s001.docx]

# Supplementary Materials

| **Table S1: PISA survey items** | |  |
| --- | --- | --- |
| Variable | Survey items | Response options |
| Home possessions^1^ | - A room of your own - A computer (laptop, desktop, or tablet) that you can use for schoolwork - Educational Software or Apps - Your own <cell phone> with Internet access (e.g., smartphone) - Internet access (e.g., Wi-fi) (excluding through smartphones) | Yes  No |
|  | - Cars, vans, or trucks - Mopeds or motorcycles - Rooms with a bath or shower - Rooms with a <flush toilet> | None  One  Two  Three or more |
|  | - Televisions - Desktop computers - Laptop computers or notebooks - Tables (e.g., <iPad>, <BlackBerry PlayBook>) - E-book readers (e.g., <Kindle, Kobo, <Bookeen>) - <Cell phones> with Internet access (i.e., smartphones) | None  1 or 2  3 or 5  More than 5 |
|  | - Books on science - <Technical reference books> - Dictionaries - Books to help with your school work | None  1-5  6-10  More than 10 |
| Arts resources^1^ | - Classical literature (e.g., <Shakespeare>) - Contemporary literature - Books on art, music, or design | None  1-5  6-10  More than 10 |
|  | - Musical instruments (e.g., guitar, piano, <country-specific example>) - Works of art (e.g., paintings, sculptures, <country-specific example>) | None  One  Two  Three or more |
| Openness to art and reflection | - I enjoy creating art - I enjoy artistic activities - I express myself through art - I reflect on movies I watch - I see beauty in everyday things | Strongly disagree  Disagree  Agree  Strongly agree |
| Creative school and classroom environment | - My teachers give me enough time to come up with creative solutions on assignments. - My teachers value students’ creativity. - The activities we do in my classes help me think about new ways to solve problems. - My teachers encourage me to come up with original answers. - At school, I am given a chance to express my ideas. | Strongly disagree  Disagree  Agree  Strongly agree |
| Note: ^1^Home possessions and arts resources only included items that were administered in every country; country-specific response items were excluded. For each of these four variables, responses were considered in the analysis if participants completed at least 80% of the items. For example, participants who answered 4 out of 5 items for a given variable were still included to maintain a good sample size. | | |

| **Table S2: Patterns and rates of in-school and out-of-school arts and creative engagement across 73 countries and regions (visually shown in Figure 1).** | | | |
| --- | --- | --- | --- |
| **Country** | **Sample size** | **School engagement** | **Outside school engagement** |
| Korea | 6,246 | 92.2% | 52.6% |
| Thailand | 8,054 | 86.4% | 78.6% |
| Philippines | 6,102 | 85.7% | 78.1% |
| Indonesia | 12,029 | 84.6% | 76.4% |
| Albania | 3,370 | 84.3% | 79.5% |
| Palestinian Authority | 5,721 | 84.0% | 77.8% |
| Chinese Taipei | 5,692 | 83.8% | 41.4% |
| Kosovo | 4,142 | 83.4% | 76.4% |
| Peru | 4,564 | 83.4% | 67.0% |
| Macao (China) | 4,262 | 82.3% | 47.2% |
| Brazil | 6,843 | 80.5% | 67.8% |
| North Macedonia | 4,511 | 80.2% | 72.5% |
| Kazakhstan | 18,373 | 79.0% | 71.3% |
| Colombia | 5,905 | 78.8% | 64.0% |
| Dominican Republic | 2,900 | 78.6% | 69.8% |
| Estonia | 5,797 | 78.5% | 51.3% |
| Baku (Azerbaijan) | 2,612 | 78.0% | 71.2% |
| Germany | 4,333 | 77.8% | 55.5% |
| Jordan | 5,137 | 77.3% | 71.2% |
| Georgia | 3,800 | 76.4% | 68.8% |
| Uzbekistan | 4,350 | 75.6% | 72.0% |
| United Arab Emirates | 19,765 | 75.1% | 64.1% |
| Malaysia | 6,030 | 74.9% | 62.9% |
| Mongolia | 5,577 | 74.7% | 60.7% |
| Romania | 5,961 | 74.2% | 62.7% |
| Morocco | 3,720 | 74.2% | 69.4% |
| Ireland | 4,996 | 73.5% | 46.1% |
| Chile | 4,543 | 73.5% | 50.8% |
| Jamaica | 1,896 | 73.4% | 57.5% |
| Türkiye | 6,860 | 71.7% | 53.5% |
| Uruguay | 3,614 | 71.3% | 54.1% |
| Switzerland | 4,617 | 70.6% | 49.0% |
| Costa Rica | 4,799 | 70.4% | 59.5% |
| Canada | 17,214 | 69.8% | 47.0% |
| Hong Kong (China) | 5,404 | 69.7% | 46.0% |
| Argentina | 6,685 | 69.5% | 56.5% |
| Panama | 1,706 | 69.3% | 61.4% |
| New Zealand | 3,716 | 69.3% | 45.6% |
| Qatar | 4,909 | 68.9% | 60.9% |
| El Salvador | 4,092 | 68.7% | 61.6% |
| Bulgaria | 4,169 | 68.4% | 62.9% |
| Iceland | 2,366 | 68.2% | 56.0% |
| Republic of Moldova | 5,104 | 68.2% | 63.4% |
| Australia | 11,940 | 68.2% | 44.6% |
| Brunei Darussalam | 4,475 | 67.5% | 51.2% |
| Ukrainian regions (18 of 27) | 2,706 | 67.0% | 58.3% |
| Saudi Arabia | 5,456 | 66.4% | 63.7% |
| Finland | 7,730 | 64.8% | 35.8% |
| Austria | 4,828 | 64.1% | 46.8% |
| Singapore | 6,438 | 63.9% | 38.4% |
| Norway | 4,949 | 63.8% | 41.9% |
| United Kingdom | 9,441 | 62.5% | 38.8% |
| Montenegro | 4,333 | 61.8% | 54.3% |
| Latvia | 4,310 | 59.6% | 50.9% |
| Mexico | 5,151 | 59.4% | 51.3% |
| Malta | 2,544 | 58.9% | 48.0% |
| Spain | 26,000 | 58.7% | 42.3% |
| Slovenia | 5,743 | 58.7% | 43.5% |
| Israel | 4,693 | 58.3% | 47.4% |
| Netherlands | 4,123 | 57.0% | 34.7% |
| Slovak Republic | 4,628 | 55.1% | 51.7% |
| Greece | 5,407 | 54.4% | 48.6% |
| Serbia | 5,007 | 52.1% | 44.7% |
| Hungary | 5,141 | 50.6% | 39.7% |
| Croatia | 5,228 | 45.9% | 40.5% |
| Denmark | 4,222 | 44.9% | 31.6% |
| Belgium | 6,015 | 43.1% | 35.8% |
| Lithuania | 6,222 | 39.2% | 37.6% |
| France | 4,845 | 38.5% | 36.0% |
| Poland | 5,156 | 35.9% | 31.9% |
| Czech Republic | 7,018 | 35.8% | 36.3% |
| Portugal | 5,782 | 35.6% | 33.8% |
| Italy | 9,166 | 34.9% | 31.7% |
| **Total** | **441,183** | **66.4%** | **53.2%** |

| **Table S3: A summary of results** | | | | | | | | | | |
| --- | --- | --- | --- | --- | --- | --- | --- | --- | --- | --- |
|  | **Main analysis^1^** | | **In high-income countries^2^** | | **In Middle-income countries^2^** | | **Omitting ‘Not available’ response^1^** | | **Restricting to Public school only^1^** | |
|  | In school | Outside school | In school | Outside school | In school | Outside school | In school | Outside school | In school | Outside school |
| ***Individual-related predictors*** |  |  |  |  |  |  |  |  |  |  |
| Female (ref male) |  |  | 🡹 |  |  | 🡻 |  |  |  | 🡻 |
| Native born (ref immigrant) | 🡻 | 🡻 | 🡻 | 🡻 | 🡻 | 🡻 | 🡻 | 🡻 | 🡻 | 🡻 |
| [Parent] Upper secondary education (ref [Parent] up to lower secondary education) |  | 🡻 | 🡻 |  |  | 🡻 |  | 🡻 |  | 🡻 |
| [Parent] Degree or above (ref [Parent] up to lower secondary education) |  |  |  |  | 🡹 | 🡹 | 🡹 |  | 🡹 | 🡹 |
| Home possessions |  | 🡻 |  | 🡻 |  | 🡻 |  | 🡻 |  | 🡻 |
| Arts resources | 🡹 | 🡹 | 🡹 | 🡹 | 🡹 | 🡹 | 🡹 | 🡹 | 🡹 | 🡹 |
| Openness to art and reflection | 🡹 | 🡹 | 🡹 | 🡹 | 🡹 | 🡹 | 🡹 | 🡹 | 🡹 | 🡹 |
| ***School-related predictors*** |  |  |  |  |  |  |  |  |  |  |
| School in town (ref school in rural area) | 🡻 | 🡻 | 🡻 | 🡻 | 🡻 | 🡻 | 🡻 | 🡻 | 🡻 | 🡻 |
| School in city (ref school in rural area) | 🡻 | 🡻 | 🡻 | 🡻 | 🡻 | 🡻 | 🡻 | 🡻 | 🡻 | 🡻 |
| Private school (ref public school)^3^ |  | 🡻 | 🡹 |  | 🡻 | 🡻 | 🡹 |  | - | - |
| Creative school and classroom environment | 🡹 | 🡻 | 🡹 |  | 🡹 | 🡻 | 🡹 |  | 🡹 | 🡻 |
| ***Country-related predictors*** |  |  |  |  |  |  |  |  |  |  |
| World happiness index |  |  | - | - | - | - |  |  |  |  |
| Gini income inequality index | 🡹 | 🡹 | - | - | - | - | 🡹 | 🡹 | 🡹 | 🡹 |
| Logged GDP in capita | 🡻 | 🡻 | - | - | - | - | 🡻 | 🡻 | 🡻 | 🡻 |
| Government expenditure in education | 🡹 | 🡹 | - | - | - | - | 🡹 | 🡹 | 🡹 | 🡹 |
| Note: ^1^Estimates from multilevel modelling. ^2^Estimates from logistic regression. Country-level predictors were omitted from the regression. ^3^ Type of school was omitted from the model restricting to public school only. | | | | | | | | | | |

**
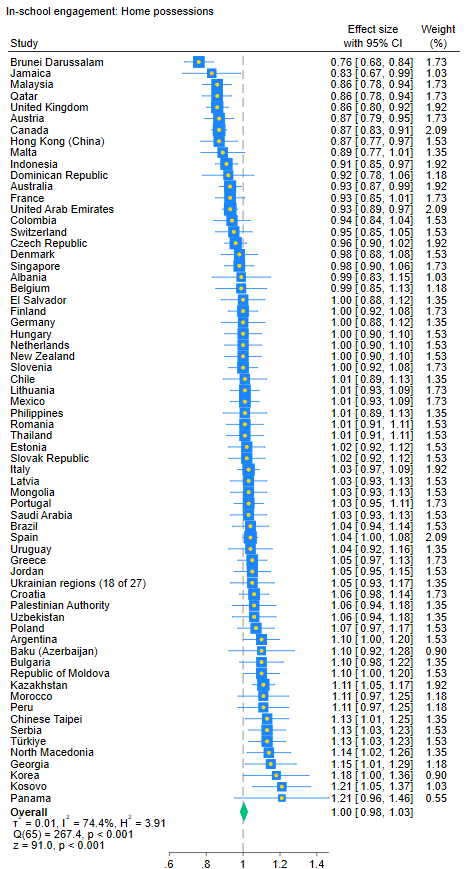
**

**Figure S1a|** Meta-analysis of the findings for home possessions and in-school engagement from logistic regression (n study= 66; sample n = 308,008). Data were first analysed separately for each country using logistic regression. The findings were then pooled into meta-analyses using the random effects model to estimate the overall effect sizes for all outcomes. Odds ratios and 95%CI are presented.

**
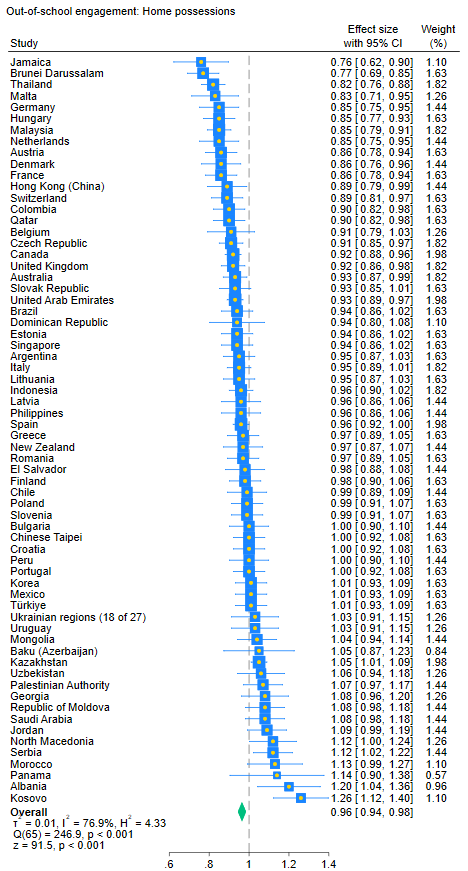
**

**Figure S1b|** Meta-analysis of the findings for home possessions and out-of-school engagement from logistic regression (n study= 66; sample n = 308,008). Data were first analysed separately for each country using logistic regression. The findings were then pooled into meta-analyses using the random effects model to estimate the overall effect sizes for all outcomes. Odds ratios and 95%CI are presented.

**
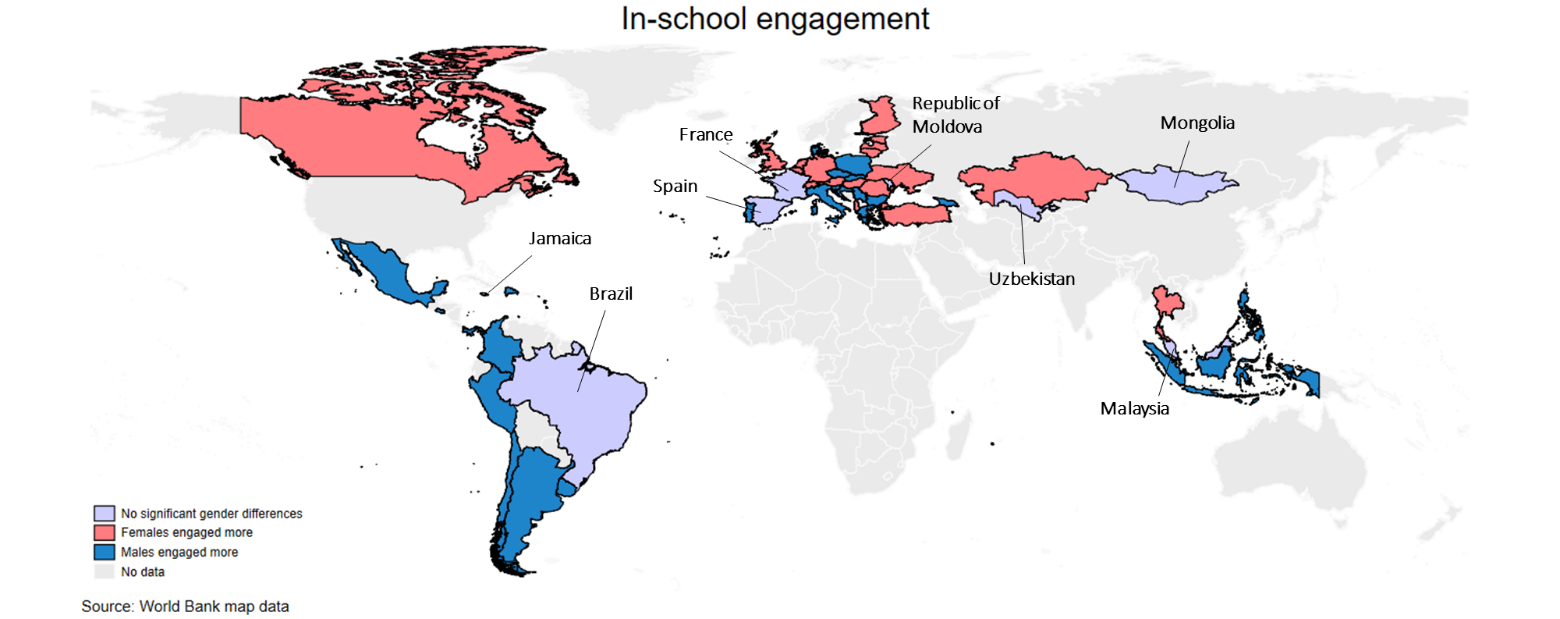
**

**
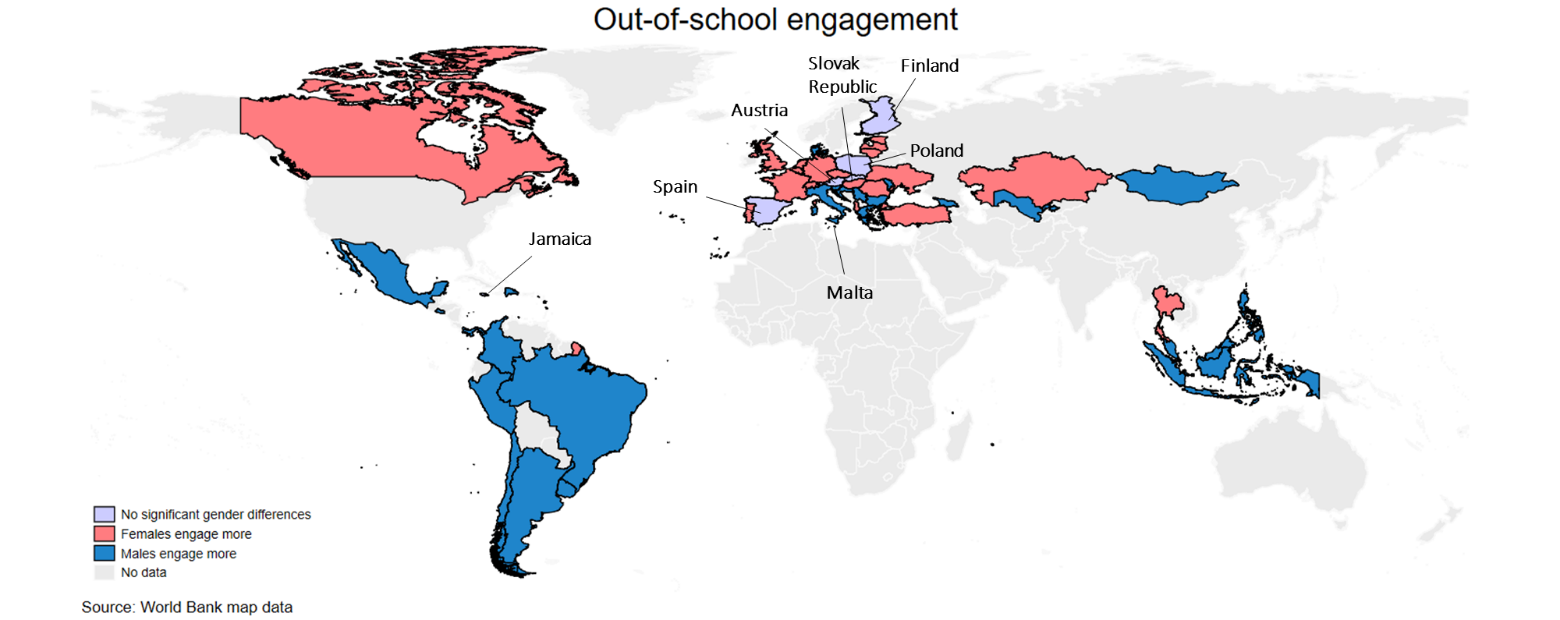
**

**Figure S2 |** Gender differences in in-school and out-of-school arts and creative engagement across 50 countries or regions (N= 234,872). Post-estimations from multilevel models. Pink countries or regions indicate that females were more likely to engage in those countries or regions; blue countries or regions indicate that males were more likely to engage; and purple countries or regions indicate no significant gender differences in engagement.

**
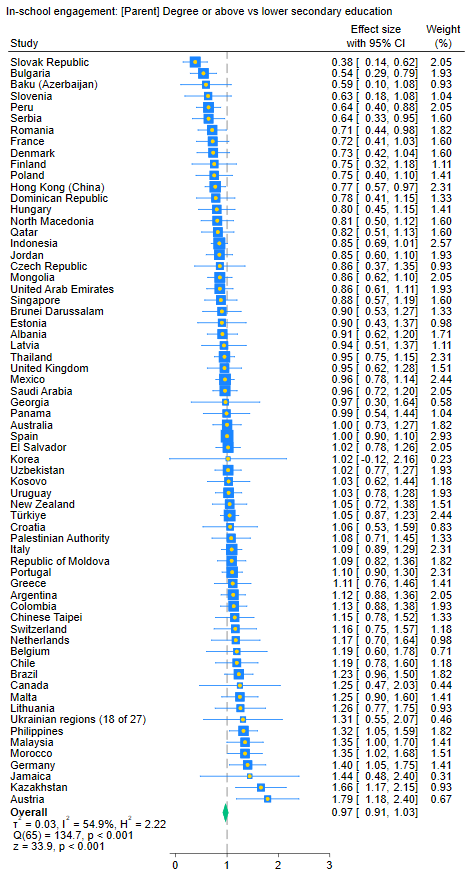
**

**Figure S3a|** Meta-analysis of the findings for parents’ education level and in-school engagement from logistic regression (n study= 66; sample n = 308,008). Data were first analysed separately for each country using logistic regression. The findings were then pooled into meta-analyses using the random effects model to estimate the overall effect sizes for all outcomes. Odds ratios and 95%CI are presented.

**
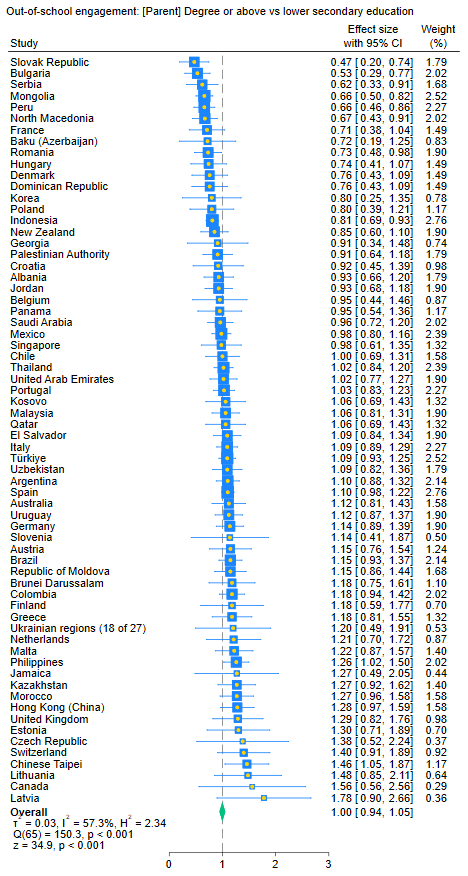
**

**Figure S3b|** Meta-analysis of the findings for parents’ education level and out-of-school engagement from logistic regression (n study= 66; sample n = 308,008). Data were first analysed separately for each country using logistic regression. The findings were then pooled into meta-analyses using the random effects model to estimate the overall effect sizes for all outcomes. Odds ratios and 95%CI are presented.

**
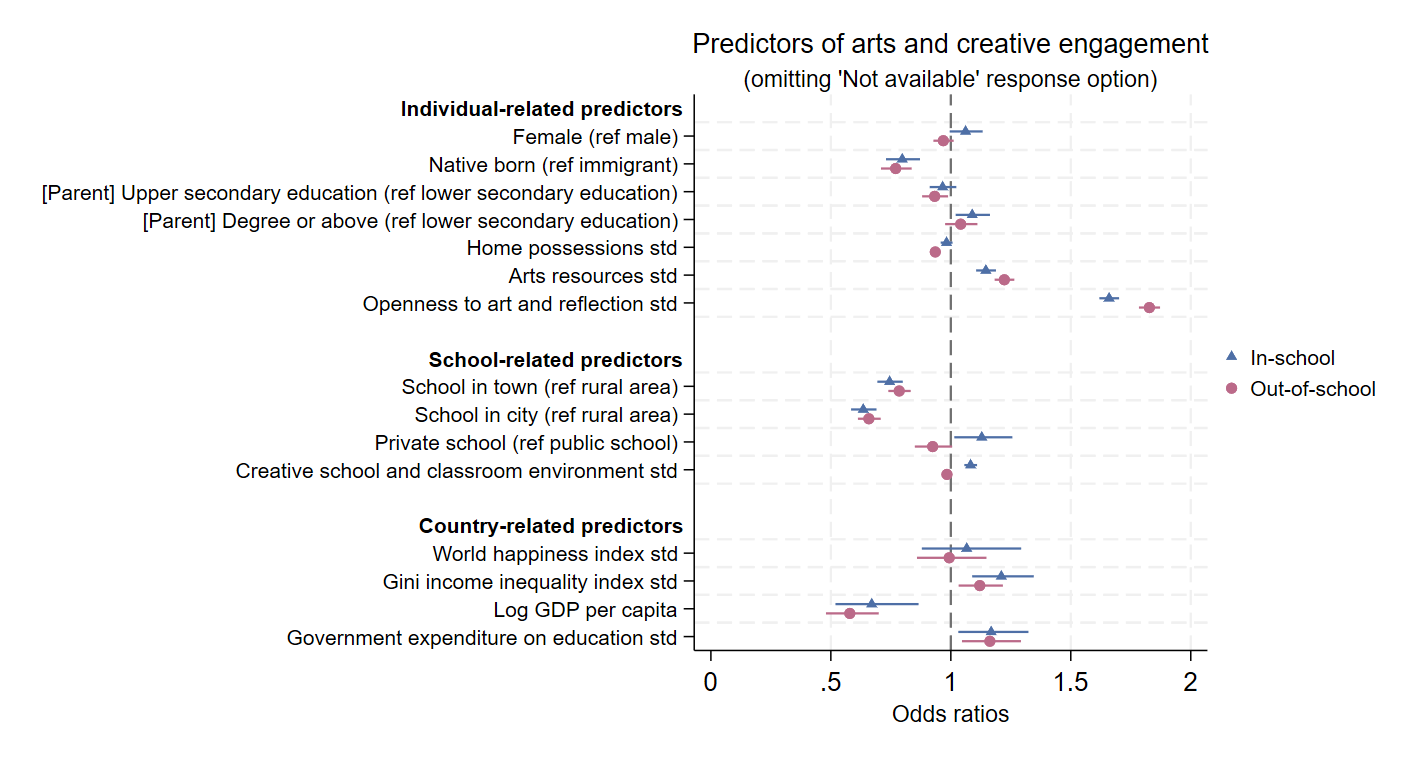
**

**Figure S4** | Predictors of in-school and out-of-school arts and creative engagement, estimates from multilevel logistic regression models (N=199,739; 50 countries or regions): omitting ‘Not available’ response item. Odds ratios and 95%CI are presented.


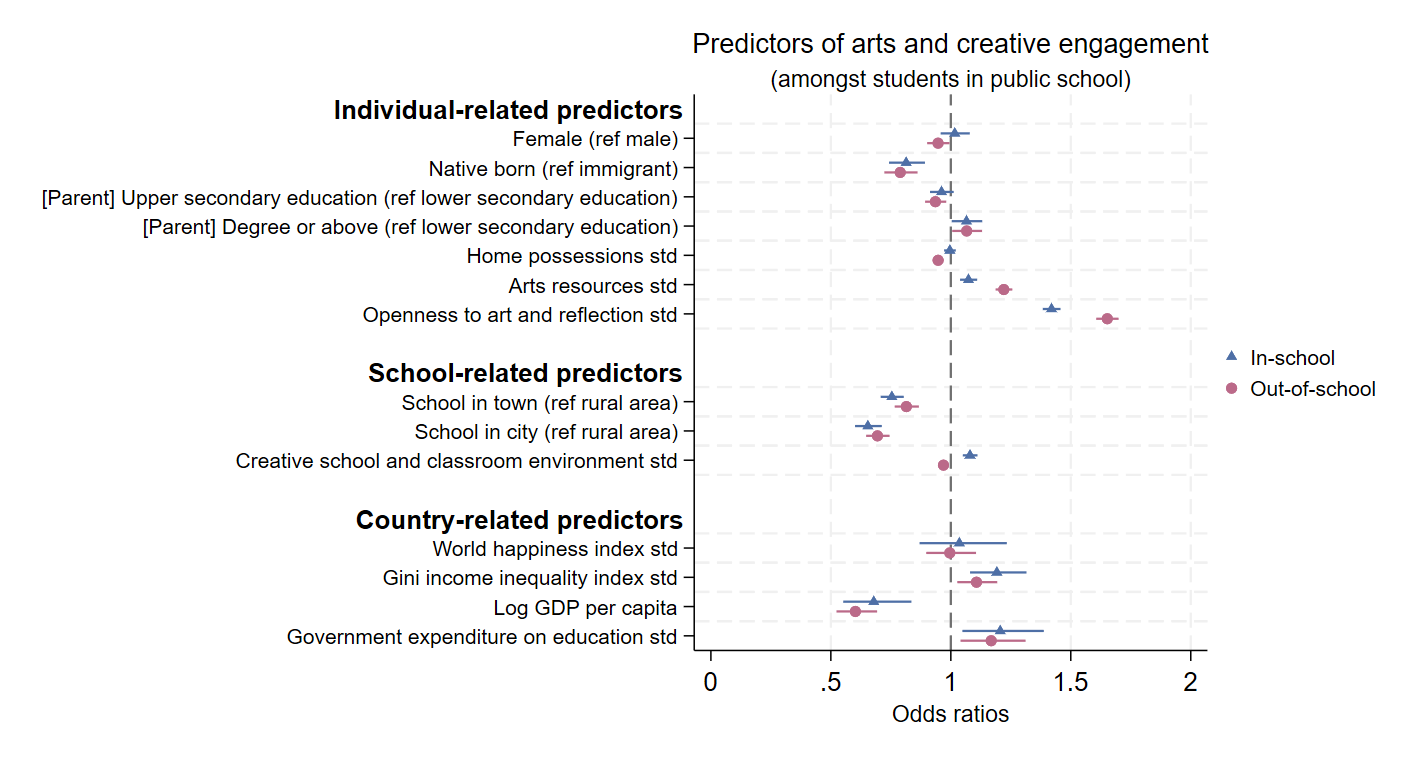


**Figure S5 |** Predictors of in-school and out-of-school arts and creative engagement, estimates from multilevel logistic regression models (N= 194,753; 50 countries or regions): restricting to students in public school. Odds ratios and 95%CI are presented.

**
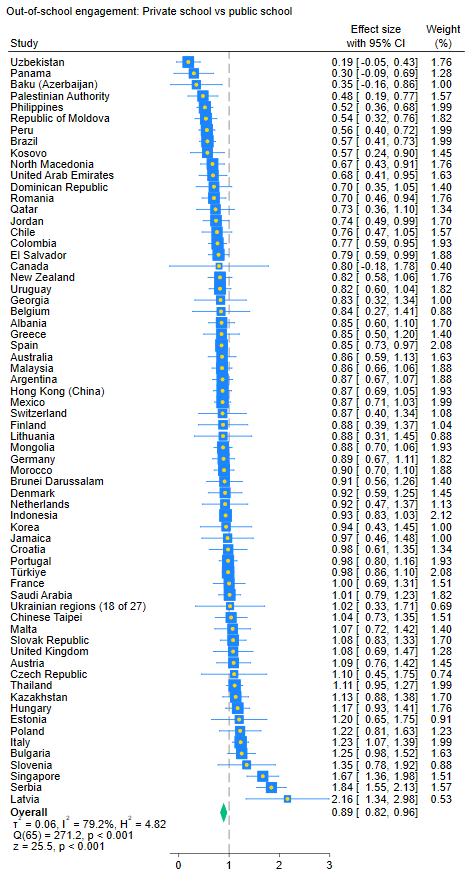
**

**Figure S6a|** Meta-analysis of the findings for school types and out-of-school engagement from logistic regression (n study= 66; sample n = 308,008). Data were first analysed separately for each country using logistic regression. The findings were then pooled into meta-analyses using the random effects model to estimate the overall effect sizes for all outcomes. Odds ratios and 95%CI are presented.

**
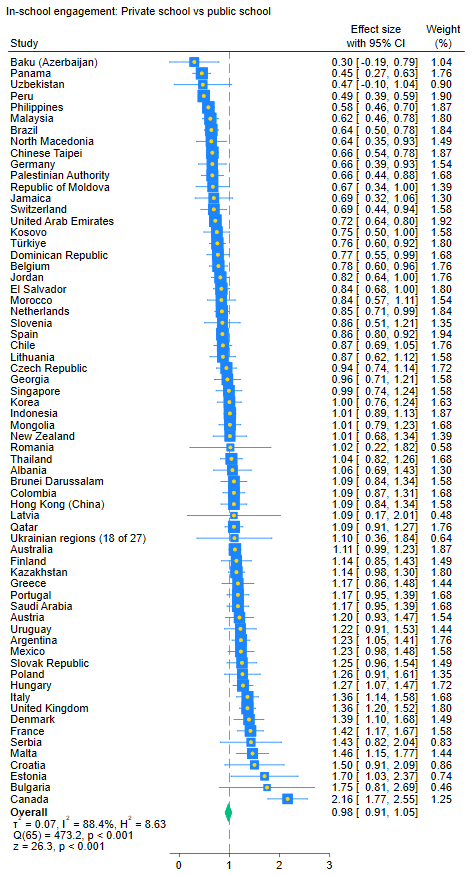
**

**Figure S6b|** Meta-analysis of the findings for school types and in-school engagement from logistic regression (n study= 66; sample n = 308,008). Data were first analysed separately for each country using logistic regression. The findings were then pooled into meta-analyses using the random effects model to estimate the overall effect sizes for all outcomes. Odds ratios and 95%CI are presented.
